# Incidence of Galactorrhea in Young Women Using Depot-Medroxyprogesterone Acetate

**DOI:** 10.1100/tsw.2006.106

**Published:** 2006-05-05

**Authors:** Hatim A. Omar, Rana M. Zakharia, Shibani Kanungo, Marlene Huff, Kimberly McClanahan

**Affiliations:** Department of Pediatrics, University of Kentucky, Lexington, USA

**Keywords:** adolescence, adolescent contraception, injectable progesterone, Depo-Provera, teenage mothers, galactorrhea, USA

## Abstract

Galactorrhea is rarely mentioned as a possible side effect of the use of Depot-Medroxyprogesterone Acetate (DMPA). Over the last few years, we have noticed an increased number of patients complaining of galactorrhea. A review of clinical data showed that between 1999 and 2005, 360 adolescents in our clinic used DMPA for at least 6 months. After medical follow-up, 13 (3.6%) of these patients were found to have developed galactorrhea. The mean age of the patients was 19.4 years with a range from 13—24. Prolactin levels in these patients were normal, and in all subjects, the galactorrhea resolved spontaneously within the next year in both patients who continued use and those who discontinued use of DMPA. It appears that galactorrhea is a benign side effect and as previous reports have suggested, it did not seem to be related to changes in Prolactin levels in our patients. It is thought that this is a progesterone-mediated effect. We believe that reassurance and education of patients is sufficient and there is no evidence of need for further intervention. Since the sample size is small in this study, additional research is recommended as to validate the presence of progesterone-mediated effects secondary to the use of DMPA.

## INTRODUCTION

Depot-Medroxyprogesterone Acetate (DMPA) or Depo-Provera has gained distinction for its benefits as well as its side effects. There have been numerous studies conducted on related clinical issues such as weight gain, bone mineral density, epithelial and mucus changes in the lower genital tract, depression, and acceptability and continuation rates, but few studies have looked at the incidence or impact of galactorrhea in patients who use DMPA. This lack of literature is in sharp contrast to the plethora of early studies looking at galactorrhea in oral contraceptive (OC) users and recommendations on its management[1,2,3,4,5,6].

There are a few studies in the literature that cite galactorrhea as a side effect associated with DMPA use[7,8,9,10]. In an early study, Powell and Seymour[8] reported that 3 of 1,123 (0.3%) adult DMPA users reported galactorrhea. In a larger study using a comparison group, Gongsakdi and Rojanasakul[10]

compared 97 adult parous DMPA users with 40 control intrauterine device (IUD) users. They found a significant difference in the incidence of galactorrhea between DMPA users and IUD users, with 64% of their DMPA sample exhibiting galactorrhea and only 15% of IUD users having the same side effect. The authors concluded that galactorrhea is a common side effect in multiparous DMPA users. They also found no difference in serum Prolactin levels (PRL) in DMPA users with and without galactorrhea, and all sella turcica X-rays performed on women with galactorrhea were normal. Thus, it was concluded that galactorrhea, while common in multiparous users of DMPA, is not clinically significant. A later report by Cromwell and Anyan[7] described DMPA use in adolescents at a hospital-based clinic from 1984 to 1992. They found that 6 out of 64 (9%) adolescents developed galactorrhea followed by spontaneous resolution with continued DMPA use. Cromwell and Anyan also found normal Prolactin levels and concluded that galactorrhea associated with DMPA use is benign. Additionally, Cromer[9], in her recent review of clinical issues related to DMPA use, noted that galactorrhea in association with the use of DMPA appears to be a benign effect.

## METHODS

A retrospective review of clinical records in regard to DMPA use and incidence of galactorrhea was completed for patients attending the Adolescent Medicine Clinic at a university-based health center in a medium-sized city. Adolescent patients who took DMPA between 1999 and 2005 for at least 6 months were included. The total sample of patients was 360. See Table 1 for demographic information.

**TABLE 1**
**Demographic Characteristics of Patients Included in the Study**

| | |
|---|---|
| Mean age | 19.4 years |
| Standard deviation | 2.2 years |
| Median | 19.5 years |
| Age range | 13–24 years |
| White | 52% |
| Black | 46% |
| Other | 2% |
| Single | 67% |
| Married | 13% |
| Cohabitating | 6% |
| Undetermined | 14% |
| Para 0 | 61% |
| Para 1 | 30% |
| Para >1 | 9% |

## RESULTS

Between 1999 and 2005, 360 adolescent patients took DMPA for at least 6 months. The average length of use in the included patient was 14 months with a range of 6–28 months. The largest percentage of patients was nulliparous (61%), 30% had one birth, and 9% had more than one birth. Thirteen (3.6%) of the sample reported having galactorrhea. Of those 13 patients, 6 (46%) patients were nulliparous, 6 (46%) had had one birth, and 1 (8%) had had more than one birth. Prolactin levels in these patients were normal, except in one nulliparous patient that had a slightly elevated Prolactin level (65 ng/ml with normal being below 20 ng/ml). This patient, however, disclosed excessive breast and nipple stimulation. Once the excessive stimulation was reduced, the Prolactin level normalized and the galactorrhea resolved 3 months later. In this patient, imaging studies were obtained and showed normal sella turcica. In all patients, the galactorrhea resolved spontaneously within the next year in both patients who continued use and those who discontinued use of DMPA**.**

## DISCUSSION

This study looked at adolescent females between the ages of 13 and 24 years who chose DMPA as their method of birth control for at least 6 months. While many other studies have studied side effects of DMPA in the areas of weight gain, bone mineral density, epithelial and mucus changes in the lower genital tract, depression, and acceptability and continuation rates[11,12], few have mentioned the side effect of galactorrhea. Of the few studies that have reported on galactorrhea, two reported on adult users of DMPA and showed a range of 0.3–64%[8,10] of patients exhibiting galactorrhea. Another study examined adolescent users and found that 9% of the subjects developed galactorrhea[7]. In our population, the incidence found in this study was 3.6%. It may have to do with the total number of patients or just simple variations between patients. As with other studies that have measured Prolactin levels in patients with galactorrhea in association with DMPA use, we found that Prolactin levels were normal in our subsample of patients who exhibited galactorrhea except in the one patient with excessive breast stimulation.

The large variability in percentage of participants exhibiting galactorrhea in association with DMPA use is interesting, particularly in the adult samples with the highest prevalence rate being 64%. It should be noted that those patients were primarily multiparous, while the other adult study (0.3%) does not inform the reader as to how many births patients had had; thus, it is hard to determine if there is any relationship between number of pregnancies and propensity toward galactorrhea in DMPA users in adults. However, given that three of the four studies reviewed here had an overall percentage of patients with galactorrhea associated with DMPA use under 10%, one must wonder if there were particular characteristics beyond parity that distinguished that sample of women with a 64% galactorrhea rate. Additionally, in contrast to the multiparous nature of the adult sample with 64% galactorrhea, in our current sample of adolescents, the majority (92%) were either Para 0 or Para 1. Thus, it does not follow that more births, at least in the adolescent sample, provides a propensity toward galactorrhea with DMPA use. Given the conflicting findings of these studies, it is difficult to determine whether parity has any effect on incidence of galactorrhea with DMPA use. If progesterone is responsible for proliferation of the acini of the mammary gland, it may be possible that repeated pregnancies, at least in adult women, may lead to a propensity for galactorrhea to occur in adults. In adolescents, at least with regard to our sample, this does not appear to be the case. While this study indicates that galactorrhea appears to be a benign side effect of DMPA use in both adolescent and adult women, its possibility as a side effect should be brought to the attention of the user of DMPA in order to mitigate any potential adverse reactions by the user of the drug and to improve compliance, especially in adolescents.

However, it may be reasonable to speculate that some difference exists between hormonal response in adolescent and adult females and, therefore, it would be reasonable to follow up with studies that might be better able to determine whether or not there is a relationship between number of births, DMPA use, and galactorrhea, and whether that pattern is the same across age groups. In addition, there have been suggestions in the literature that DMPA as progesterone is responsible for proliferation of the acini of the mammary gland and persists with continuing stimulation of the breast by the individual, partner, or clothing, which helps explain the patient discussed above. More research is needed to study this assertion and to better explain this effect of DMPA.

## BIOSKETCHES

**Hatim A. Omar, MD,** Professor of Pediatrics and Obstetrics and Gynecology and Director of the Section of Adolescent Medicine, Department of Pediatrics, University of Kentucky, Lexington. Dr. Omar has completed residency training in obstetrics and gynecology as well as pediatrics. He has also completed fellowships in vascular physiology and adolescent medicine. He is the recipient of the Commonwealth of Kentucky Governor's Award for Community Service and Volunteerism and is well known internationally with numerous publications in child health, pediatrics, adolescent medicine, and pediatric and adolescent gynecology. E-mail: haomar2@uky.edu.

**Rana M. Zakharia,** Undergraduate Student, University of Kentucky, Lexington. E-mail: rmzakh1@uky.edu

**Shibani Kanungo, MD,** Resident Pediatrics, University of Kentucky, Lexington. E-mail: skanu2@email.uky.edu

**Marlene B. Huff, PhD**, is an Associate Professor of Pediatrics at the Kentucky Clinic, University of Kentucky, Lexington. She has publications in the area of disability studies and various aspects of clinical social work. E-mail: marlene.huff@uky.edu

**Kimberly MaClanahan, PhD,** Assistant Professor, Section of Adolescent Medicine, Department of Pediatrics, University of Kentucky, Lexington. E-mail: kimberly.mcclanahan@uky.edu
